# Ultrasonic Spray-Coating of Large-Scale TiO_2_ Compact Layer for Efficient Flexible Perovskite Solar Cells

**DOI:** 10.3390/mi8020055

**Published:** 2017-02-14

**Authors:** Peng Zhou, Wangnan Li, Tianhui Li, Tongle Bu, Xueping Liu, Jing Li, Jiang He, Rui Chen, Kunpeng Li, Juan Zhao, Fuzhi Huang

**Affiliations:** 1 State Key Laboratory of Advanced Technology for Materials Synthesis and Processing, Wuhan University of Technology, Wuhan 430070, China; whutzhp@gmail.com (P.Z.); litianhui@whut.edu.cn (T.L.); tonglebu@whut.edu.cn (T.B.); xuepingliu@whut.edu.cn (X.L.); lijing123@whut.edu.cn (J.L.); milijiang@whut.edu.cn (J.H.); Rui-Chen@whut.edu.cn (R.C.); likunpeng@whut.edu.cn (K.L.); 2 Hubei Key Laboratory of Low Dimensional Optoelectronic Materials and Devices, Hubei University of Arts and Science, Xiangyang 441053, China; wangnan.li@yahoo.com; 3 School of Automotive Engineering, Wuhan University of Technology, Wuhan 430070, China

**Keywords:** ultrasonic spray, titanium oxide, flexible perovskite solar cells, low temperature, large area

## Abstract

Flexible electronics have attracted great interest in applications for the wearable devices. Flexible solar cells can be integrated into the flexible electronics as the power source for the wearable devices. In this work, an ultrasonic spray-coating method was employed to deposit TiO_2_ nanoparticles on polymer substrates for the fabrication of flexible perovskite solar cells (PSCs). Pre-synthesized TiO_2_ nanoparticles were first dispersed in ethanol to prepare the precursor solutions with different concentrations (0.5 mg/mL, 1.0 mg/mL, 2.0 mg/mL) and then sprayed onto the conductive substrates to produce compact TiO_2_ films with different thicknesses (from 30 nm to 150 nm). The effect of the different drying processes on the quality of the compact TiO_2_ film was studied. In order to further improve the film quality, titanium diisopropoxide *bis*(acetylacetonate) (TAA) was added into the TiO_2_-ethanol solution at a mole ratio of 1.0 mol % with respect to the TiO_2_ content. The final prepared PSC devices showed a power conversion efficiency (PCE) of 14.32% based on the indium doped tin oxide coated glass (ITO-glass) substrate and 10.87% on the indium doped tin oxide coated polyethylene naphthalate (ITO-PEN) flexible substrate.

## 1. Introduction

With the global energy consumption increasing, cheap, green and environmentally friendly energy sources are in urgent demand due to the reduction of fossil fuels. Photovoltaic technology is an ideal solution to alleviate the energy crisis and environmental pollution problems. Organic-inorganic lead halide perovskite solar cells (PSCs) have attracted great interest due to their rapid increase in power conversion efficiencies (PCE) from 3.8% to 22.1% within only seven years [1,2,3]. These great improvements are mainly attributed to the excellent photo-electronic properties of perovskite materials, such as high light absorption properties, direct bandgaps, high charge-carrier mobility and a long electron–hole exciton transport distance (more than 1 μm) [4,5,6]. Compared to the commercial silicon-based solar cells, PSCs show great advantages with a simplified architecture and a low-cost solution-processed technology, which give them great potential for the future photovoltaic industry [7].

Organic-inorganic lead halide perovskite (CH_3_NH_3_PbI_3_) was first used as a sensitizer in dye-sensitized solar cells (DSSCs) by Kojima in 2009 [8] with a PCE of 3.8%, but the performance decreased rapidly due to the dissolution of the perovskite in the liquid electrolytes. Two years later, the solid-state hole-transporting material 2,2′,7,7′-tetrakis-(N,N-di-p-methoxypheny-lamine) 9,9′-spirobifluorene (spiro-OMeTAD) was introduced by Park and Grätzel, and achieved a reported PCE of 9.7% [9]. Since the all solid-state-type PSCs were fabricated, the photo-electronic performance improved rapidly with the use of different electronic/hole transporting semi-conductive materials, such as TiO_2_, ZnO, SnO, PCBM, P_3_HT, etc. [10]. Depending on the different architectures, the PSCs can be generally classified into mesoscopic, meso-superstructured and planar heterojunction types [11]. Among these types, the planar PSCs have a simplified architecture and are easily produced by a solution process. In a planar solar cell, the photoactive layer, CH_3_NH_3_PbI_3_, is sandwiched between an electron-transporting layer (ETL) and a hole-transporting layer (HTL), which is suitable for large-scale commercial manufacturing layer-by-layer. The ETL plays an important role as it allows the transport of electrons while blocking the holes. Thus, it influences the carriers’ injection, collection, transportation, recombination, and then the overall performance of the PSCs [12]. Anatase TiO_2_ is the most widely used ETL for planar PSCs, but it still requires a high temperature sintering of the TiO_2_ compact layer to achieve a high efficiency. In general, this compact layer is prepared by spin-coating or spray pyrolysis of a TiO_2_ precursor solution with subsequent sintering at 500 °C to transform the amorphous oxide layer into the crystalline phase (anatase), which provides good charge transport properties [13]. The involvement of the high temperature sintering process of TiO_2_ has limited the development of flexible PSCs fabricated on plastic substrates, such as polyethylene terephthalate (PET) and polyethylene naphthalate (PEN).

Since the first flexible perovskite solar cell was reported [14], more efforts have been devoted to it and have achieved efficiencies of over 10% on polymer substrates [15,16], demonstrating that efficient perovskite solar cells can be fabricated at low temperatures with a “regular” design. Atomic layer deposition [17,18], microwave sintering [19] and inductively coupled plasma (ICP)-assisted DC magnetron sputtering [20] have been used for deposition of the TiO_2_ compact layer at low temperatures. A photonic-cured compact TiO_2_ layer has been used on a PET substrate with a high efficiency of 11.2% by Xiao [21]. Snaith et al. realized flexible PSC with an efficiency of 15.9% on a low-temperature processed TiO_2_ compact layer by spin-coating the TiO_2_ precursor on fluorine doped tin oxide coated glass (FTO-glass) [22]. Yang et al. developed a process to fabricate a very dense amorphous TiO_2_ using DC magnetron sputtering at room temperature and achieved 15.07% PCE based on a flexible PET substrate [23]. Sanjib et al. used a combination of ultrasonic spray-coating and a low-thermal-budget photonic curing technology for the first time to fabricate a flexible PSC with an efficiency of 8.1% [24]. Supasai et al. fabricated compact layers of crystalline TiO_2_ thin films using aerosol spray pyrolysis on FTO-glass for PSCs, achieving the best efficiency of 6.24% [25]. Kim et al. fabricated a mesoporous TiO_2_ ETL with a large area of 10 cm × 10 cm using electro-spray deposition (ESD) for the first time, which resulted in an optimized PCE of 15.11%, higher than that (13.67%) of the PSC with spin-coated TiO_2_ films [26]. Both the aerosol spraying and ESD methods demonstrated great potential in the large-scale fabrication of PSCs. However, the TiO_2_ films deposited by the above two spraying methods require high temperature sintering, which is not suitable for the preparation of flexible PSCs. The use of these technologies has allowed for the development of all-low-temperature processed PSCs on flexible substrates, but at the same time, has made the process more complicated, resulting in the increase of the manufacturing cost. 

Here, we report a low temperature fabrication (<150 °C) of a compact layer composed of highly crystalline small nanoparticles of anatase TiO_2_ (diameter <10 nm) dispersed in ethanol. The ultrasonic spray-coating method was employed for the deposition of the TiO_2_ compact layer, demonstrating the capability to precisely and reliably deposit thin and uniform layers. Various parameters have to be considered in the process of ultrasonic spraying, such as the flow rate of the TiO_2_ ethanol solution, the gas flow pressure which carries the sprayed droplets to the substrate, the distance between the spray nozzle and the substrate, as well as the moving speed of the nozzle during spraying [27,28]. Through the optimization of these parameters, two technological regimes named “wet-film” (w-film) and “dry-film” (d-film) are compared in our work. The former approach results in a dense TiO_2_ layer of different thicknesses (30 nm to 150 nm) by changing the concentration of the dispersion. In addition, a small amount of titanium diisopropoxide *bis*(acetylaceto-nate) (TAA) (1.0 mol % with respect to the TiO_2_ content) is added to the colloidal TiO_2_ dispersion, resulting in the highest PCE of 14.32% based on ITO-glass and 10.87% based on ITO-PEN, both with an active area of 0.4 cm × 0.4 cm. Through the same method, a large-area flexible PSC of 5 cm × 5 cm is fabricated.

## 2. Materials and Methods

### 2.1. Materials

Unless specified otherwise, all the chemicals were purchased from either Alfa Aesar or Sigma-Aldrich and used as received. 2,2′,7,7′-tetrakis-(N,N-di-p-methoxypheny-lamine) 9,9′-spirobifluorene (spiro-OMeTAD) was purchased from Shenzhen Feiming Science and Technology Co., Ltd. (Shenzhen, China), and MAI (CH_3_NH_3_I) was purchased from Lumtec, Taiwan.

### 2.2. Substrates

Indium doped tin oxide coated glass (ITO-Glass) and polyethylene naphthalate (ITO-PEN) were etched by zinc powder and hydrochloric acid, then followed by ultrasonic cleaning in detergent, pure water and ethanol for 15 min, respectively. The substrate (ITO-Glass or ITO-PEN) was cut into suitable size and plasma cleaned for 5 min to remove any organic material on the surface. Especially, the flexible ITO-PEN substrate was mounted onto glass micro-slides before using.

### 2.3. Synthesis of TiO_2_ Nanoparticles

The anatase titanium oxide nanoparticles were synthesized according to previously reported method published elsewhere [29]. Briefly, 0.5 mL of anhydrous TiCl_4_ (99.9%, Aladdin, Shanghai, China) was added dropwise into 2 mL of anhydrous ethanol (Sigma-Aldrich, Shanghai, China), stirring for 5 min till the mixed yellow liquid was cooled down to room temperature. Then the whole content was transferred into a vial containing 10 mL anhydrous benzyl alcohol (Aladdin, Shanghai, China), with the color of the clear solution changing to light yellow. The solution was heated to 80 °C and reacted for 9 h. After the reaction the solution was cooled down to the room temperature, and a translucent dispersion of very fine TiO_2_ nanoparticles was obtained. Then add 36 mL of diethyl ether to 4 mL of the above solution to precipitate the TiO_2_ which was centrifuged at 4000 rpm for 5 min, washed with ethanol and diethyl ether. The above steps were repeated three times and the final precipitation was redispersed in 10 mL of anhydrous ethanol, resulting in a colloidal solution of approximately 12 mg TiO_2_/mL ethanol but easily aggregated. The TiO_2_ ethanol solution was further diluted into 0.5 mg/mL, 1.0 mg/mL and 2.0 mg/mL respectively for the late ultrasonic spraying. In order to disperse the TiO_2_ nanoparticles and enhance the adhesion as well as the connection between nanoparticles, a small amount of TAA was added into the diluted dispersion with a mole ratio of 1.0 mol %, 2.0 mol % and 3.0 mol % respectively. The solution was left to stand for at least 2 h before spraying, but could be stable for months.

### 2.4. Deposition of the TiO_2_ Compact Layer

**Figure 1:**
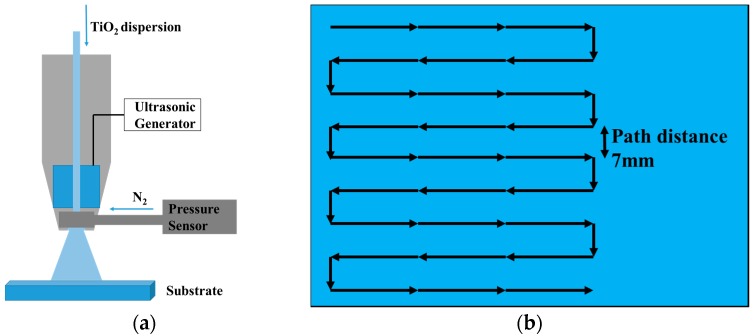
Schematic representation of (**a**) setup for the spray coater and (**b**) the spray nozzle moving path.

The low-temperature processed TiO_2_ compact layer was deposited by ultrasonic spraying the colloidal dispersion of anatase particles in anhydrous ethanol, formulated with TAA, followed by treating at 135 °C for 1 h to remove ethanol solvent. Figure 1a shows the schematic representation of setup for the spray coating. Figure 1b showed the spray nozzle moving path during the spray. Spray coating was carried out in an Exacta Coat Ultrasonic Spraying System (Sono-Tek Corpration, Milton, NY, USA) equipped with an AccuMist nozzle. The thickness of the compact layer was tuned by the concentration of TiO_2_ nanoparticles (0.5–2.0 mg/mL). 

### 2.5. Device Fabrication

After deposition of the TiO_2_ compact layer, the substrate with appropriate size (1.5 cm × 1.5 cm for small devices and 5.0 cm × 5.0 cm for large area devices) was transferred into a N_2_ filled glove box. The perovskite and hole conductor solutions were prepared in a N_2_ glove box before deposition. The perovskite was deposited by spin coating 25 μL of a 1.25 mol/L solution of CH_3_NH_3_I and PbI_2_ (molar ratio 1:1) in DMF at 6500 rpm for 30 s using the gas-assisted method [30], meanwhile a 60 psi dry N_2_ gas stream was blown onto the film for 8 s from the third second of spinning. For the fabrication of flexible devices with large area, 200 μL perovskite solution was coated on TiO_2_ compact layer and spin-coated at 5000 rpm for 30 s, followed by dropping 200 μL chlorobenzene (CBZ) at the fifth second of spinning. The films were subsequently annealed on a hot plate at 100 °C for 10 min in the glove box. After letting the films cool for 5 min, 20 μL spiro-OMeTAD/CBZ solution (68 mM spiro-OMeTAD, 150 mM tert-butylpyridine and 25 mM lithium *bis*(*tri*-fluoromethanesulphonyl)imide) was spin-coated at 3000 rpm for 30 s. Promptly after the hole transport material deposition, a gold counter electrode (60 nm) was evaporated under high vacuum to complete the device.

### 2.6. Characterizations

The morphologies and microstructures of the prepared low-temperature processed TiO_2_ compact and the cross-sectional structure of the perovskite solar cells were investigated using a field-emission scanning electron microscopy (FE-SEM, Zeiss Ultra Plus). To examine the surface roughness, the films were characterized by BY2000 atomic force microscopy (AFM). The TiO_2_ anatase phase was tested by an X-ray diffractometer (XRD, D8 Advance). The photocurrent density-voltage curves of the PSCs were measured using a solar simulator (Oriel 94023A, 300 W). The intensity (100 mW/cm^2^) was calibrated using a standard Si-solar cell (Oriel, VLSI standards). All the devices were tested under AM 1.5 G sun light (100 mW/cm^2^) using a metal mask of 0.16 cm^2^ with a scan rate of 0.01 V/s. Some parameters would be mentioned to evaluate the performance of PSCs, such as open circuit voltage (*V*_oc_), short circuit current (*J*_sc_), fill fact (FF), and power conversion efficiency (PCE).

## 3. Results and Discussion

**Figure 2:**
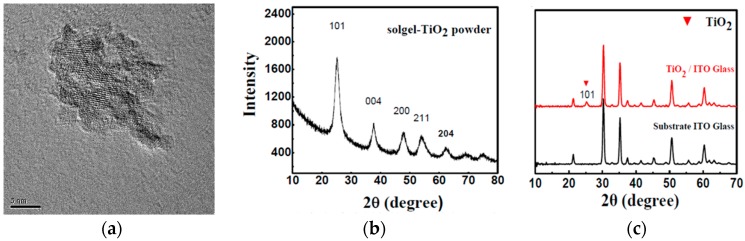
(**a**) Transmission electron microscopy (TEM) image of the TiO_2_ nanoparticles dispersed in ethanol at a concentration of 1.0 mg/mL; (**b**) XRD pattern of the TiO_2_ powder drying at 135 °C; and (**c**) XRD patterns of ITO-glass with/without the TiO_2_ compact layer coated by the ultrasonic spraying.

Anatase-type TiO_2_ nanoparticles were synthesized through a method published elsewhere [29], using titanium tetrachloride (TiCl_4_) as the precursor, and anhydrous ethanol and benzyl alcohol as solvents. The TiO_2_ nanoparticles synthesized via this method are highly crystalline, shown in Figure 2a,b. In the XRD pattern of the TiO_2_ powder prepared by drying the as-synthesized nanoparticles at 135 °C to evaporate the ethanol solvent, three typical diffraction peaks occur at 25.5°, 38.5° and 48°, which respectively belong to the (101), (004) and (200) planes of the anatase TiO_2_ crystal. The as-synthesized TiO_2_ nanoparticles could easily be dispersed in ethanol, which can be used for ultrasonic spraying directly. After the deposition of the TiO_2_ layer, the films had to be treated at 135 °C for 1 h, replacing the high-temperature sintering process. The size of the TiO_2_ nanoparticles was around 5 nm, as shown in the TEM image (Figure 2a), which can also be calculated by the Scherrer equation using the (101) diffraction peak through the formula reported elsewhere [31]. Figure 2c shows the XRD patterns of ITO-glass with/without the TiO_2_ layer, in which just a weak diffraction peak of TiO_2_ occurs at 25.5° due to the thin thickness of the TiO_2_ layer. 

In order to obtain uniform and dense films of TiO_2_ compact layers, two technological regimes of ultrasonic spraying were built. During ultrasonic spraying, many parameters influence the uniformity, roughness, and coverage of the TiO_2_ film, such as the flow rate of the TiO_2_ ethanol solution, the gas flow pressure which carries the sprayed droplets to the substrate, the distance between the spray nozzle and the substrate, as well as the moving speed of the nozzle. The ultrasonic spraying technology was employed in the deposition of polymer films such as polyvinylpyrrolidone (PVP) by Sanjukta Bose et al. in 2013 [27]. Parts of the parameters have been discussed, resulting in uniform organic films with small roughness compared to the thickness of the films. Based on the work mentioned above, we optimized the process of spraying, and employed it to deposit inorganic TiO_2_ film. Figure 1a shows the schematic representation of the setup for the spray-coating. Figure 1b shows the ultrasonic spraying trajectory over the substrate. Snaith et al. realized low-temperature processed PSCs with power convention efficiencies (PCE) of up to 15.9% using a spin-coating method, demonstrating the optimum thickness of TiO_2_ to be approximately 45 nm [22], while the thickness of TiO_2_ prepared by high-temperature sintering at 500 °C is around 30 nm [7]. Based on these reports, we tuned the thickness of ultrasonic spraying TiO_2_ compact layer ranging from 30 nm to 100 nm by changing the spraying parameters and the concentration of the TiO_2_ ethanol solution. 

**Figure 3:**
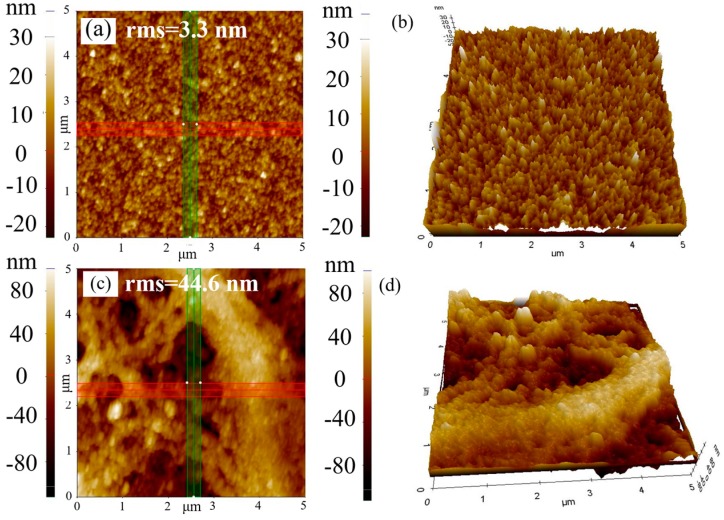
Atomic force microscope (AFM) images of the surface of the TiO_2_ layers sprayed by (**a**,**b**) the w-film; (**c**,**d**) the d-film using the TiO_2_ ethanol solution without TAA.

During the spraying, two different technological regimes were termed “wet-film” (w-film) and “dry-film” (d-film), depending on the drying type of the ethanol solvent. The former regime means that the ethanol solvent evaporates after the droplets are sprayed onto the substrate, forming a very uniform thin liquid membrane layer before the ethanol evaporates. A few seconds later, the solvent begins to evaporate, resulting in a uniform thin compact layer of TiO_2_. The latter regime means that the processing of ethanol evaporation occurs mainly before the droplets are sprayed onto the substrate, without forming the liquid membrane. The TiO_2_ compact layer prepared by the former regime was influenced by the “coffee-ring” effect, which is described by Deegan et al. as the result of capillary flow [32], where liquid evaporates faster from the pinned contact line of a deposited solution and is replenished by additional liquid from inside; however, this did not happen in the latter regime. During the spraying of the w-film regime, the pressure of the N_2_ flow was set to 0.3 psi, the flow rate of solution was set to 1.2 mL/min, and the speed of the moving nozzle was set to 50 mm/s, which can result in a thin liquid membrane on the surface of the substrate before the solvent evaporates; during the d-film regime, the three parameters were respectively set to 1.0 psi, 0.3 mL/min, and 20 mm/s. Figure 3 showed the AFM surface images of the TiO_2_ compact layers by ultrasonic spraying using 1.0 mg/mL of the TiO_2_ (without TAA) ethanol solution. The TiO_2_ film prepared by the w-film regime presented a root-mean-square (RMS) surface roughness of around 3.3 nm, while it increased to 44.6 nm rapidly with the d-film regime. It was attributed to the big holes between the TiO_2_ nanoparticles prepared through the d-film regime. In the film of the d-film regime, the connection between the particles was so weak that big holes appeared because of the evaporation of ethanol before the droplets were deposited on the substrate. In addition, more spraying cycles were needed to obtain the optimum thickness of the TiO_2_ film through the d-film regime, which increased the roughness of film, while just one cycle was enough to achieve the thickness with the w-film regime. Thus, it is better to fabricate the TiO_2_ compact layer using the w-film regime.

**Figure 4:**
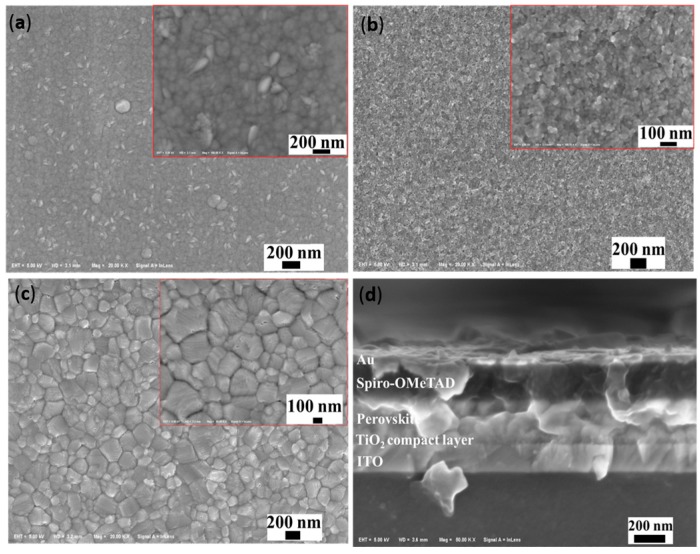
Scanning electron microscopy (SEM) image of (**a**) PEN-ITO substrate; (**b**) TiO_2_ compact layer; (**c**) perovskite prepared by the gas-assisted method; and (**d**) cross-section SEM image of the PSCs.

**Figure 5:**
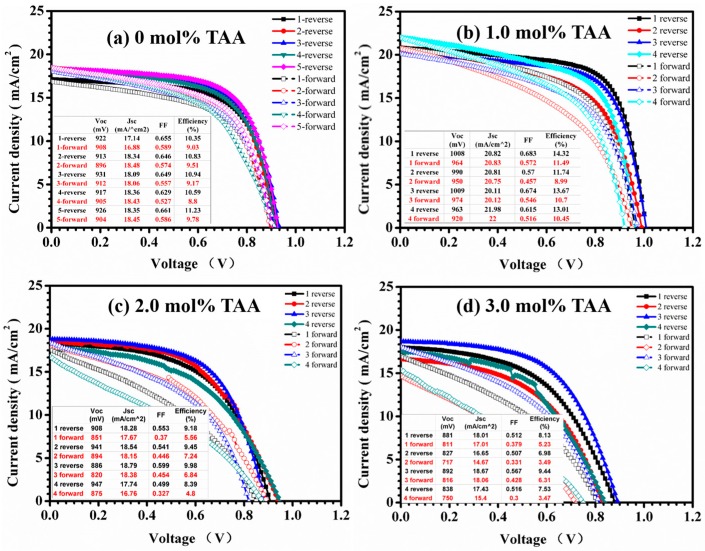
J-V characteristics of the gas-assisted processed PSCs based on TiO_2_ compact layer deposited by the ultrasonic spray-coating of TiO_2_ solution with the addition of TAA (**a**) 0 mol %; (**b**) 1.0 mol %; (**c**) 2.0 mol %; (**d**) 3.0 mol % with respect to the TiO_2_ content.

In order to discuss the influence of the thickness of the ultrasonically sprayed TiO_2_ compact layer on the performance of PSC devices, ITO-glass substrates coated with TiO_2_ films of different thicknesses (30–100 nm) were made into complete planar PSC devices. The thickness of the TiO_2_ film was tuned by changing the concentration. Table 1 shows the photovoltaic parameters obtained from the current-voltage measurements of devices with different compact layers. It is obvious that the optimal concentration for the performance was 1.0 mg/mL, with a thickness around 60 nm. Figure 4 shows the surface image of different functional layers, and a dense TiO_2_ compact layer was obtained (Figure 4b). Each functional layer can be clearly distinguished in the cross-section scanning electron microscopy (SEM) image (Figure 4d). The clear interface between the TiO_2_ layer and perovskite layer confirmed that the TiO_2_ layer was not utilized as a mesoscopic scaffold. It can be concluded that the uniform TiO_2_ compact layer with low roughness is suitable for the preparation of perovskite (Figure 4c), and the optimal thickness ranges around 60 nm. Figure 5a shows the current-voltage curves of PSCs prepared with a TiO_2_ solution of 1.0 mg/mL using the w-film regime, with an average PCE of 10.79%, and the highest PCE of 11.23%. 

**Figure 6:**
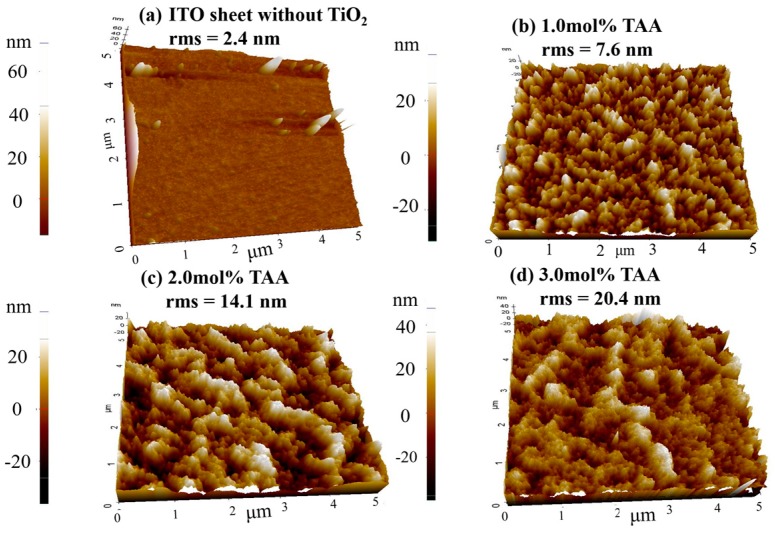
AFM images of the surface of the TiO_2_ layers sprayed by the TiO_2_ ethanol solution with the addition of TAA, (**a**) ITO sheet without TiO_2_ film; (**b**) 1.0 mol % TAA; (**c**) 2.0 mol % TAA; and (**d**) 3.0 mol % TAA with respect to the TiO_2_ content.

In addition, more works have been done to improve the performance of TiO_2_ by adding a small amount of TAA (1.0 mol %, 2.0 mol %, 3.0 mol % with respect to the TiO_2_ content), with almost a negligible influence on the thickness of the TiO_2_ film. The solutions with different amounts of TAA were ultrasonically sprayed on the substrates but with a similar thickness of the TiO_2_ compact layers, around 60 nm. The RSM increased to 7.6 nm from 3.3 nm without a significant change of the roughness of the bare ITO after 1.0 mol % TAA was added to a TiO_2_ solution of 1.0 mg/mL (Figure 6). It was found that the RMS increased with the additional increase of TAA. Furthermore, the increasing size of the large aggregates on the surface of the TiO_2_ film was observed in the AFM images (Figure 6).

**Figure 7:**
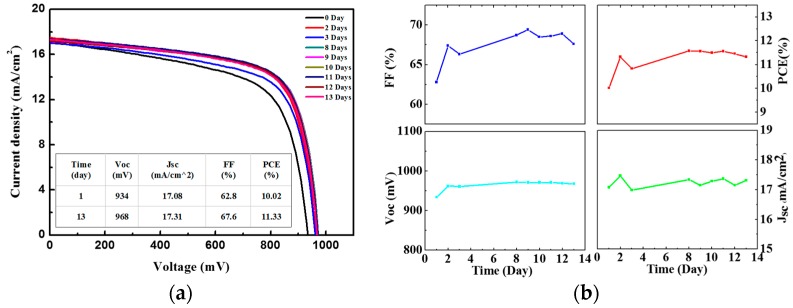
Long-duration stability test of a typical PSC based on ITO-glass, measured under AM 1.5 G sunlight (100 mW/cm^2^) using a metal mask of 0.16 cm^2^. (**a**) J-V curve of the PSC; and (**b**) FF, PCE, *J*_sc_, *V*_oc_.

The devices with the highest performance were prepared based on the TiO_2_ compact layer sprayed using the TiO_2_ solution with 1.0 mol % of TAA. The highest PCE of 14.32% was achieved, with a *V*_oc_ of 1.008 mV, a *J*_sc_ of 20.82 mA/cm^2^, and a FF of 68.3%. The improvement of the performance was attributed to the addition of TAA, which enhanced the connection of the TiO_2_ nanoparticles. However, the addition of TAA increased the roughness of the film, which had a negative influence on the preparation of the perovskite. It was also observed that the hysteresis of the PSCs increased with the addition of TAA (Figure 5). To study the stability of PSCs based on ultrasonically sprayed TiO_2_, a typical PSC on ITO-glass was placed in a N_2_-filled glove box for long-duration stability testing. The PSC showed a good stability, as shown in Figure 7. The PCE of the device increased after two days, which can be attributed to the increase of the FF, caused by the enhancement of the connection between the TiO_2_ particles and the oxidation of the Spiro-OMeTAD.

**Figure 8:**
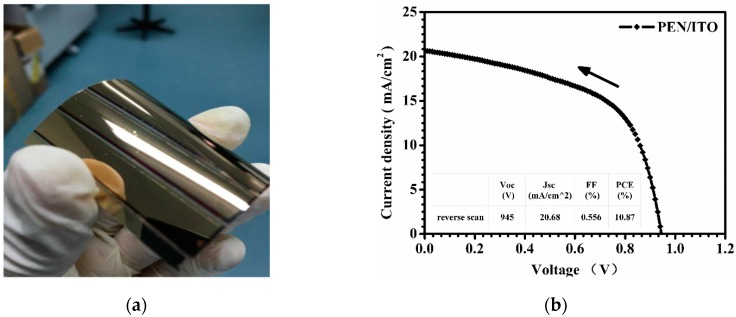
(**a**) Photographic image of the flexible device with a large area of 5.0 cm × 5.0 cm and (**b**) J-V curve of the flexible device fabricated by using the optimized parameters of ultrasonic spraying on polymer ITO-PEN substrate, tested under AM 1.5 G sunlight (100 mW/cm^2^) using a metal mask of 0.16 cm^2^.

**Figure 9:**
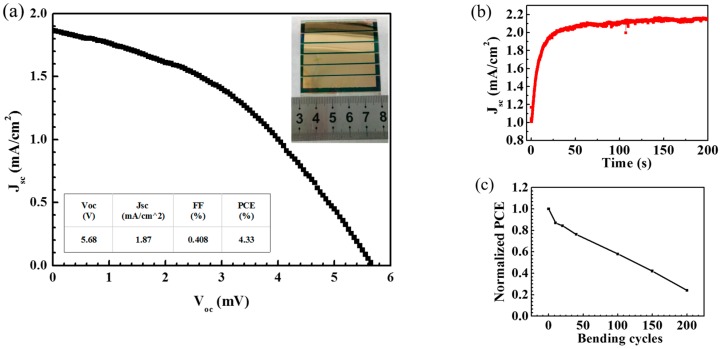
(**a**) J-V characteristics of a typical flexible PSC of 5 cm × 5 cm with an active area of 13.5 cm^2^, tested without a mask. Inset photograph is the typical flexible PSC. (**b**) Photocurrent density measured as a function of time for the same flexible PSC held at 2.5 V forward bias. (**c**) Normalized PCE of the large-area flexible PSC after bending tests using 50 mm radii of curvature.

The optimized process of the ultrasonic spraying of 1.0 mg/mL TiO_2_ solution with an addition of 1.0 mol % TAA was employed to fabricate PSCs on the flexible ITO-PEN substrate. A photographic image of the flexible device with a large area of 5.0 cm × 5.0 cm is shown in Figure 8a. The best-performing cell exhibited an outstanding photovoltaic performance with a *V*_oc_ of 945 mV, a *J*_sc_ of 20.68 mA/cm^2^, a FF of 55.6% and a PCE of 10.87%, tested under AM 1.5 G sunlight (100 mW/cm^2^) using a metal mask of 0.16 cm^2^ (Figure 8b), which offers great potential for flexible PSCs with a large area. Figure 9 shows the current-voltage curve of a flexible large-area PSC with an active area of 13.5 cm^2^, tested without a mask under standard sunlight. A PCE of 4.33% was achieved for the large-area flexible PSC.

## 4. Conclusions

In summary, using an ultrasonic spraying technology, we prepared a low-temperature processed TiO_2_ compact layer with low roughness compared to the thickness of the TiO_2_ film on both rigid and flexible substrates with a large area. Two different drying process-related regimes were studied. The spraying method of the w-film regime showed advantages in preparing low-temperature processed TiO_2_, resulting in a film with a RSM of 3.3 nm. A small amount of TAA was added into the TiO_2_ dispersion to enhance the connection of the particles, thus improving the performance of the PSCs. The best-performing device was achieved with a PCE of 14.32% based on ITO-glass, and a PCE of 10.87% based on ITO-PEN. This method provides a potential route for the fabrication of efficient large-area flexible PSCs.

## Figures and Tables

**Table 1:** The performance of devices fabricated based on the w-film ultrasonically sprayed TiO_2_ with different thicknesses tuned by the concentration of TiO_2_ dispersion.

Concentration of TiO_2_ (mg/mL)	Average Thickness of TiO_2_ Layer (nm)	*V*_oc_ (mV)	*J*_sc_ (mA/cm^2^)	FF (%)	PCE (%)
0.5	30	654	18.74	0.38	4.61
1.0	60	896	17.57	0.69	10.82
2.0	100	838	15.72	0.59	7.81
